# Influence of the initial microbiota on eggplant *shibazuke* pickle and eggplant juice fermentation

**DOI:** 10.1128/spectrum.00464-24

**Published:** 2024-07-17

**Authors:** Kazunori Sawada, Takuji Yamada

**Affiliations:** 1 Innovation Division, Gurunavi, Inc., Hibiya Mitsui Tower, Chiyoda-ku, Tokyo, Japan; 2 School of Life Science and Technology, Tokyo Institute of Technology, Meguro-ku, Tokyo, Japan; University of Torino, Torino, Italy

**Keywords:** vegetable fermentation, spontaneous fermentation, artificial microbiota, lactic acid bacteria, microbial ecosystem

## Abstract

The present study aimed to investigate the effects of the initial microbiota on microbial succession and metabolite transition during eggplant fermentation. Samples of traditional Japanese eggplant pickles, *shibazuke*, which were spontaneously fermented by plant-associated microbiota, were used for the analysis. Microbiota analysis indicated two successional patterns: early dominance of lactic acid bacteria superseded by aerobic bacteria and early dominance of lactic acid bacteria maintained to the end of the production process. Next, *shibazuke* production was modeled using filter-sterilized eggplant juice, fermenting the average composition of the initial *shibazuke* microbiota, which was artificially constructed from six major species identified during *shibazuke* production. In contrast to *shibazuke* production, all batches of eggplant juice fermentation showed almost identical microbial succession and complete dominance of *Lactiplantibacillus plantarum* in the final microbiota. These findings revealed the fate of initial microbiota under *shibazuke* production conditions: the early dominance of lactic acid bacteria that was maintained throughout, with *L. plantarum* ultimately predominating the microbiota. Furthermore, a comparison of the results between *shibazuke* production and eggplant juice fermentation suggested that *L. plantarum* is involved in the production of lactic acid, alanine, and glutamic acid during eggplant fermentation regardless of the final microbiota.

**IMPORTANCE:**

The findings shown in this study provide insight into the microbial succession during spontaneous pickle fermentation and the role of *Lactiplantibacillus* plantarum in eggplant pickle production. Moreover, the novel method of using filter-sterilized vegetable juice with an artificial microbiota to emulate spontaneous fermentation can be applied to other spontaneously fermented products. This approach allows for the evaluation of the effect of specific initial microbiota in the absence of plant-associated bacteria from raw materials potentially promoting a greater understanding of microbial behavior in complex microbial ecosystems during vegetable fermentation.

## INTRODUCTION

Pickles prepared by lactic acid fermentation are produced worldwide. Lactic acid fermentation is initiated by plant-associated bacteria or starter lactic acid bacteria (LAB) (1). Pickles using plant-associated bacteria are produced by fostering anaerobic conditions and optimizing salinity and temperature to support the growth of LAB. Although LAB initially constitute a small population within the plant-associated microbiota, they are sufficiently abundant to eventually dominate the microbiota by the end of fermentation process (2). Lactic acid fermentation by LAB is an industrially important technology because LAB enhance the nutrient value and desirable flavor while also prolonging the shelf-life of vegetables (3). This has promoted the investigation of microorganisms relevant to pickle production since the early 1900 s (4). Recent studies have aimed to provide a better understanding of the diversity and dynamics of microbial compositions during spontaneous pickle fermentation. Various fermented pickles have been used to study microbiota through 16S rRNA gene sequencing, with different genera of LAB, such as *Fructilactobacillus*, *Lacticaseibacillus*, *Lactiplantibacillus*, *Lactobacillus*, *Latilactibacillus*, *Levilactobacillus*, *Limosilactobacillus*, *Secundilactobacillus*, *Leuconostoc*, *Weissella*, and *Pediococcus*, being found to be dominant depending on the fermented products (3, 5). Studies of the well-known fermented pickles sauerkraut and kimchi have revealed the succession of the dominant LAB from *Leuconostoc* to the genus formerly annotated as *Lactobacillus* during the fermentation process (6, 7). It has been suggested that the dominance of *Lactobacillus* in the microbiota is facilitated by *Leuconostoc*, which exhibits a shorter lag phase for growth and is sensitive to acidic conditions. By producing lactic acid, *Leuconostoc* reduces the pH creating an environment that optimizes the conditions for the growth of acid-tolerant LAB, including *Lactobacillus* that produces substantial amounts of lactic acid (8). However, 16S rRNA gene sequencing has also shown that microbiota in fermented pickles can contain undesirable Gram-negative bacteria that might impair food quality and safety (9–12). Typically, Gram-negative bacteria, commonly identified as Enterobacteriaceae, are sensitive to salt and acid. However, these bacteria can remain viable in vegetable fermentation until the pH becomes acidic (13, 14). As a result, while spontaneously fermented pickles are generally free of such undesirable bacteria, inconsistent production procedures can still lead to their contamination (12). The diversity of microbiota in pickles produced by spontaneous fermentation is derived from the different raw materials, ingredients, and processes undertaken (15–17). Moreover, the microbes present during vegetable fermentation can be so sensitive to production conditions that different batches of the same pickle can harbor different microbiota (12). Given previous reports that the relative abundances of microbes influence the taste and flavor of pickles, it is important to achieve the steady succession of microbiota during spontaneous vegetable fermentation to ensure pickle quality (5, 18–21). Whereas the importance of steady succession of microbiota is well recognized, previous studies have not sufficiently explored the complexities of these ecosystems. Although numerous studies have evaluated major LAB isolated from vegetable fermentations for use as starters, the potential of inoculating specific and trackable microbiotas into sterile raw materials to replicate spontaneous fermentation processes, which can deepen our understanding of these complex ecosystems, has largely been overlooked.

**Fig 1:**
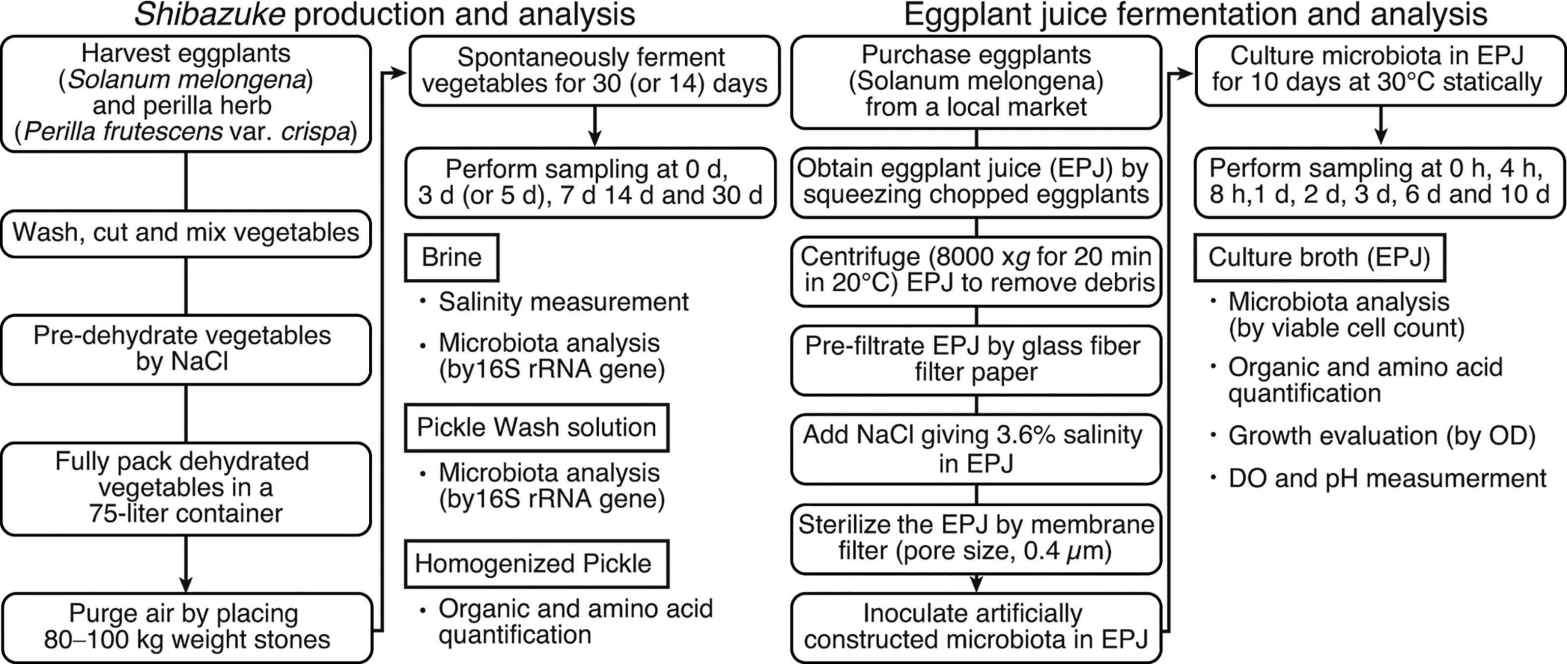
Overview of *shibazuke* production and eggplant juice fermentation. An arrow indicates the flow of the procedures. A bullet indicates the analysis performed using the samples shown in a square above. D, day(s); h, hour(s); OD, optical density; DO, dissolved oxygen.

The present study aimed to investigate the dynamics of microbiota and the relationship between microbiota and metabolites produced during pickle fermentation using a traditional Japanese fermented eggplant pickle, *shibazuke* samples (Table 1). The overview of the study is shown in Fig. 1. The findings revealed large differences in microbial succession among the production batches. Thus, *shibazuke* production was modeled to evaluate the specific effect of the initial microbiota on the succession of the microbial population by separating the microbiota from its raw material, which is usually a source of initial microbiota. Filter-sterilized eggplant juice (EPJ) was fermented by an artificially constructed microbiota designed to imitate the average initial microbiota of *shibazuke* production (Fig. 1). This model showed complete dominance of *Lactiplantibacillus plantarum*. In addition, correlation analysis and alpha-diversity analysis indicated the robust relationship between *L. plantarum* and lactic acid, alanine, and glutamic acid production. This study evaluated the effect of inoculating initial microbiota, previously identified as one of the key influencers of microbial succession, into sterile raw materials. The findings contribute to the understanding of spontaneous vegetable fermentation as a complex ecosystem. Additionally, the methods developed in this study enable the replication of these ecosystems, thereby enhancing the consistency and quality of pickle production.

**TABLE 1:** Commercial *shibazuke* pickle samples used in this study^*b*^

Sample set	Manufacturer	Sample count					
		0 d	3 d	5 d	7 d	14 d	30 d
#1 #2 #3 #4	A B C A	2^*a*^ 2^*a*^ 2^*a*^ 2^*a*^	0 1 1 1	1 0 0 0	1 1 1 1	1 1 1 1	3 1 0 3

^*a*^ It consisted of one pickle and one brine sample; d, day(s).

^*b*^ Sample set three did not have samples from 30 d because Manufacturer C finalized production in 14 d.

## RESULTS

### Salinity and pH conditions for commercial *shibazuke* production

**Fig 2:**
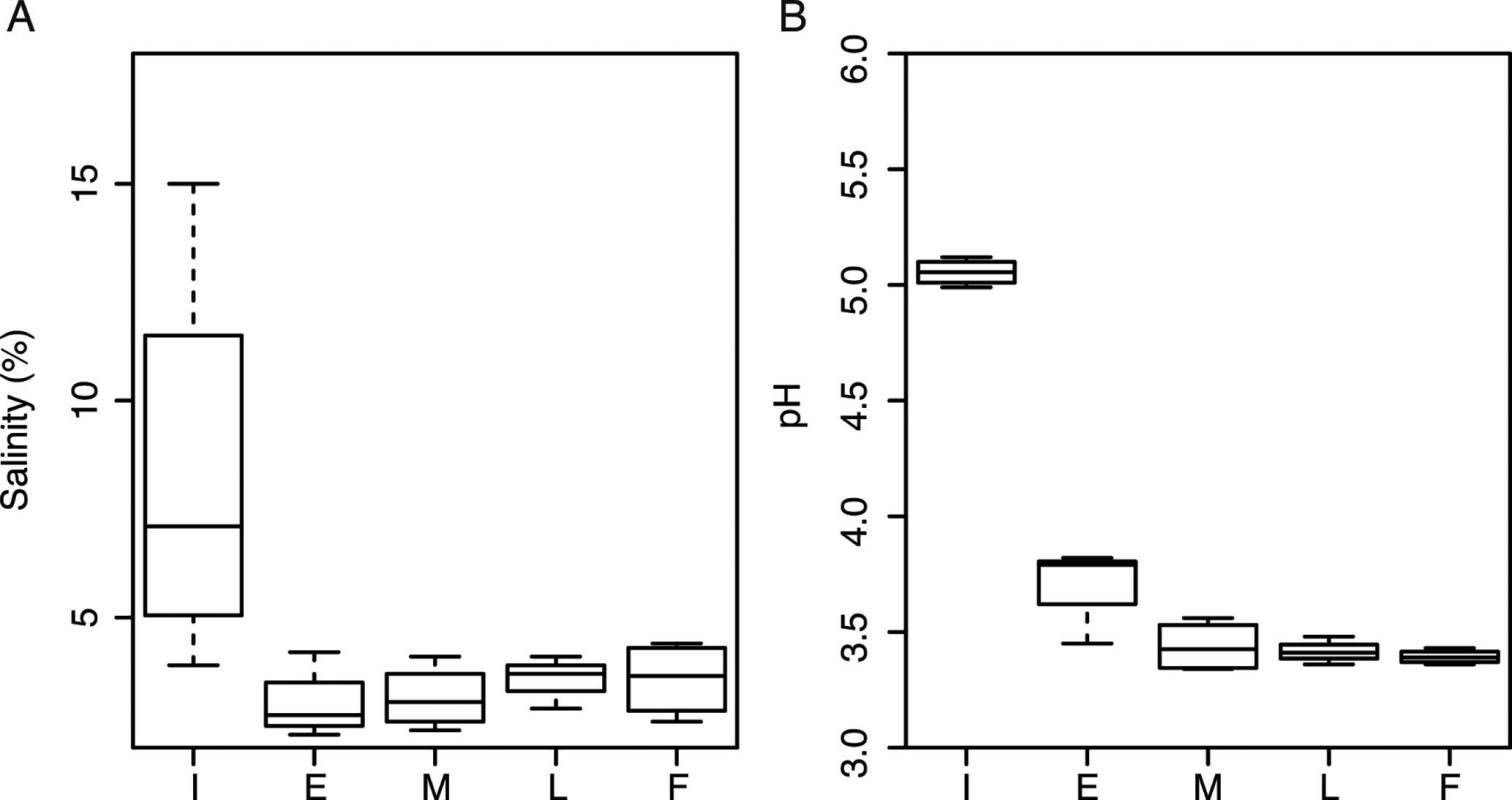
Transition of salinity and pH during the production of commercial *shibazuke* pickles. (**A**) Salinity; (**B**) pH. I, initial production [0 day (d)]; E, early production (3 or 5 d); M, mid-production (7 d); L, late production (14 d from sample set #1, 2, and 4); F, final production (30 d from sample set #1, 2, and 4, and 14 d from sample set #3). The sample of sample set 3 from 14 d was used as the final production sample as described in the manuscript. Average values were calculated from independent samples (*n* = 4) obtained from the same production point, except for late production (*n* = 3).

The salt content and pH were measured to estimate the pickling conditions during the commercial production of *shibazuke* (Fig. 2). The initial salinity showed larger variation among manufacturers, but the final salinity remained consistent. These results suggested that all manufacturers added initial salt to obtain final salinity approximately 2.5%–4.5%, and the large variation of initial salinity indicated that the salinity may not be equilibrated at the beginning of the production in fully packed containers. The variation in pH remained stable from the initial to the final product. The final pH reached 3.39 ± 0.03, which was lower than that of kimchi (pH 4.5) (22) and equivalent to sauerkraut (pH 3.4) (19).

### Changes in microbiota during commercial *shibazuke* production

**Fig 3:**
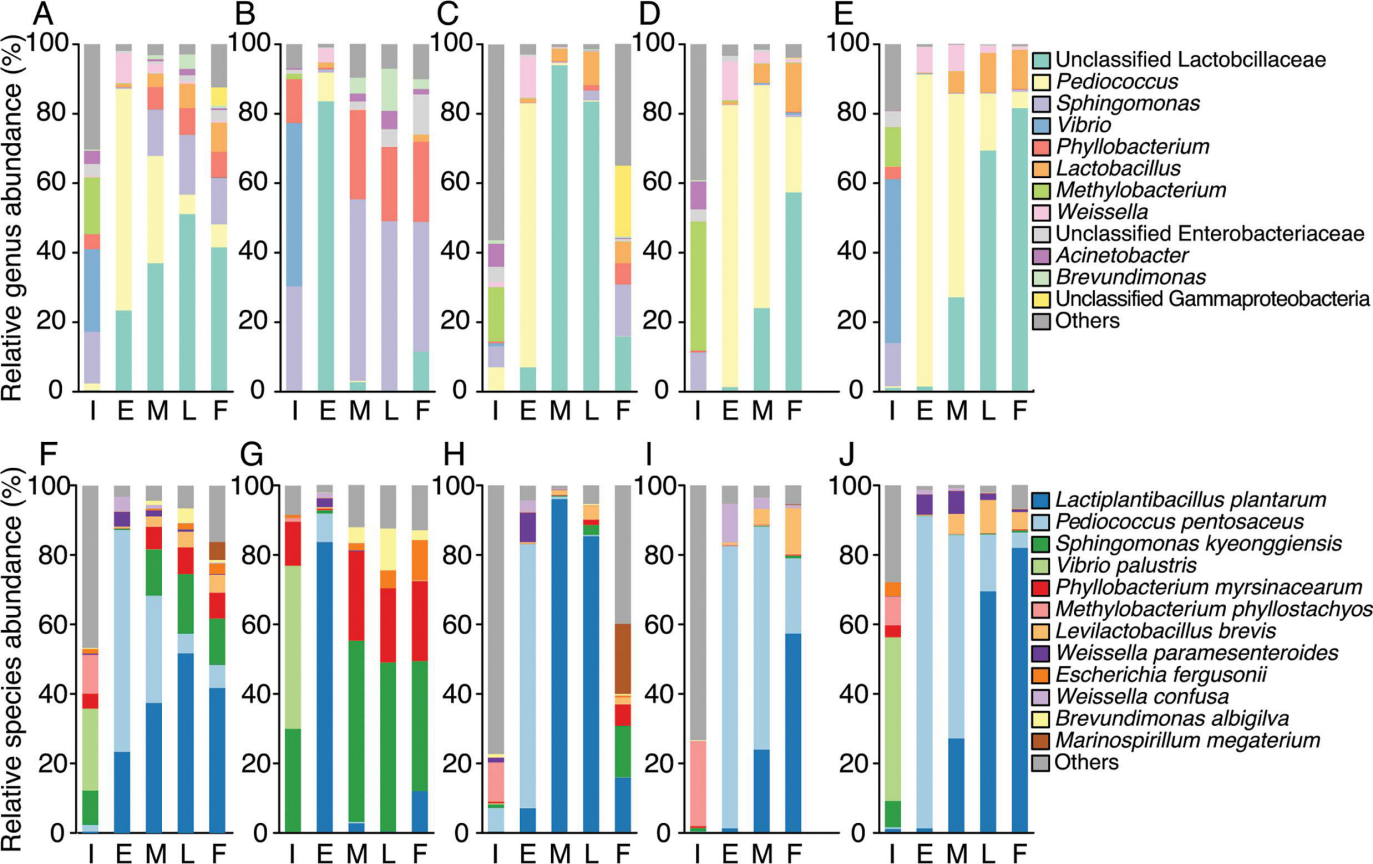
Microbial succession during the production of commercial *shibazuke* pickles. (A–E) Relative abundance of microbiota at the genus level. Genera with an average relative abundance of less than 1% were grouped into “Others.” (**A**) Average; (**B**) sample set 1; (**C**) sample set 2; (**D**) sample set 3; (**E**) sample set 4. (F–J) relative abundance of microbiota at the species level. Species with an average relative abundance of less than 1% were grouped into “Others.” (**F**) Average; (**G**) sample set 1; (**H**) sample set 2; (**I**) sample set 3; (**J**) sample set 4. I, initial production [0 day (d)]; E, early production (3 or 5 d); M, mid-production (7 d); L, late production (14 d from sample set #1, 2, and 4); F, final production (30 d from sample set #1, 2, and 4, and 14 d from sample set #3). The sample of sample set 3 from 14 d was used as the final production sample as described in the manuscript. Average values were calculated from independent samples (*n* = 4) obtained from the same production point, except for late production (*n* = 3).

The microbiota during commercial production of *shibazuke* was evaluated using 16S rRNA gene sequencing. The total and average number of reads after eliminating mitochondria and chloroplasts were 4,653,036 and 172,335 ± 115,359, respectively, from 27 samples (Table 1), among which there were 1,828 amplicon sequence variants (ASVs) and 383 genera based on the Greengenes database annotation. The average relative abundance of genera at each sampling point is shown in Fig. 3A. The microbiota at the initial production timepoint were highly diverse and included several genera of aerobic bacteria. The early production timepoint was dominated by LAB, such as *Pediococcus*, unclassified *Lactobacillaceae*, *Lactobacillus*, and *Weissella*. Unclassified *Lactobacillaceae* were relatively more abundant at the late production timepoint, but the relative abundance of LAB gradually decreased to the final production timepoint. The relative abundances of *Sphingomonas* and *Phyllobacterium* increased from late production to final production. Annotation of the ASVs at the species level showed that 12 species exhibited a relative abundance of more than 1% during production on average (Fig. 3F) and that most of the unclassified *Lactobacillaceae* were *L. plantarum*. Although the average microbiota dynamics are as shown above, it should be noted that the final microbiota in sample sets 1 and 2 were dominated by non-LAB (Fig. 3B, C, G, and H), whereas those in sample sets 3 and 4 were dominated by LAB with minor species (Fig. 3D, E, I, and J). These inconsistent results were similar to those of a previous study, which indicated that the microbiota from the same type of pickles can differ between samples due to the variations in raw material, initial microbiota, or production environments (12).

### Changes in metabolite concentrations of commercial *shibazuke* pickles

**Fig 4:**
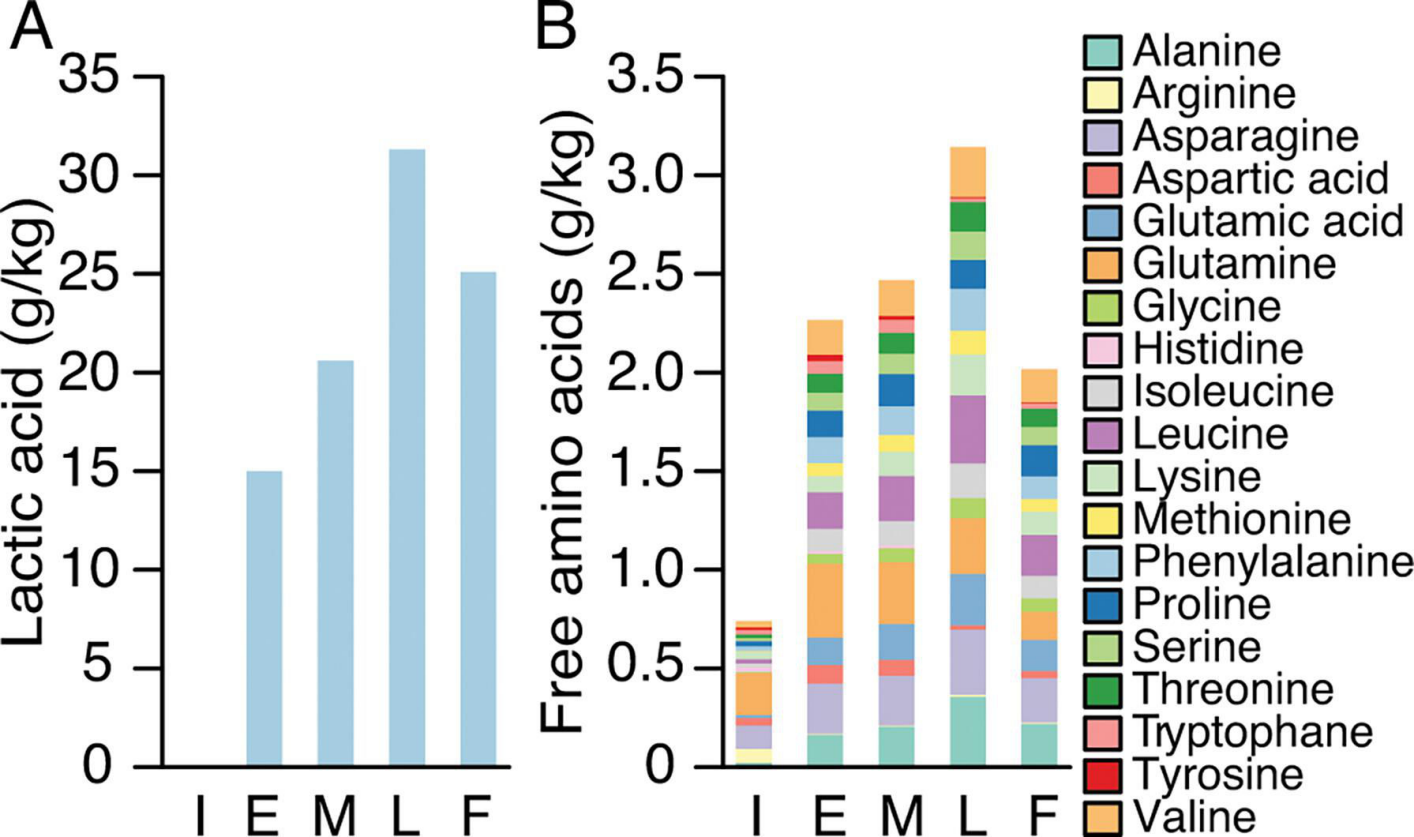
Transition of metabolite content during the production of commercial *shibazuke* pickles. (**A**) Lactic acid concentration in samples. (**B**) Free amino acids in samples. I, initial production [0 day (d)]; E, early production (3 or 5 d); M, mid-production (7 d); L, late production (14 d); F, final production (30 d). The late production sample of sample set 3 was used as the final production sample as described in the manuscript. Average values were calculated from independent samples (*n* = 4) obtained from the same production point, except for late production (*n* = 3).

The consequences of the microbial succession in *shibazuke* pickles were evaluated by quantifying the contents of metabolites. Upon high-performance liquid chromatography (HPLC) analysis of organic acids, only lactic acid was detected. The average lactic acid concentration increased until the late production timepoint, and then decreased by the final production timepoint, as shown in Fig. 4A. Despite this overall trend, lactic acid production in sample sets 3 and 4 showed an increase as detailed in Fig. S1 for each batch. The decrease in average lactic acid concentration seemingly had a negligible impact on the pH value (Fig. 2B). The results of ultra-high-performance liquid chromatography (UPLC) analysis targeting 19 amino acids are shown in Fig. 4B (Fig. S2 for each batch). Compared to the initial production timepoint, the concentrations of most amino acids increased until the late production timepoint. However, the concentrations of arginine, histidine, and tyrosine decreased to trace levels (<10 mg/kg) after the late production timepoint, which may inhibit the growth of auxotrophic lactic acid bacteria that require these amino acids.

### Fermentation profiles of the EPJ model using the artificially constructed microbiota

**Fig 5:**
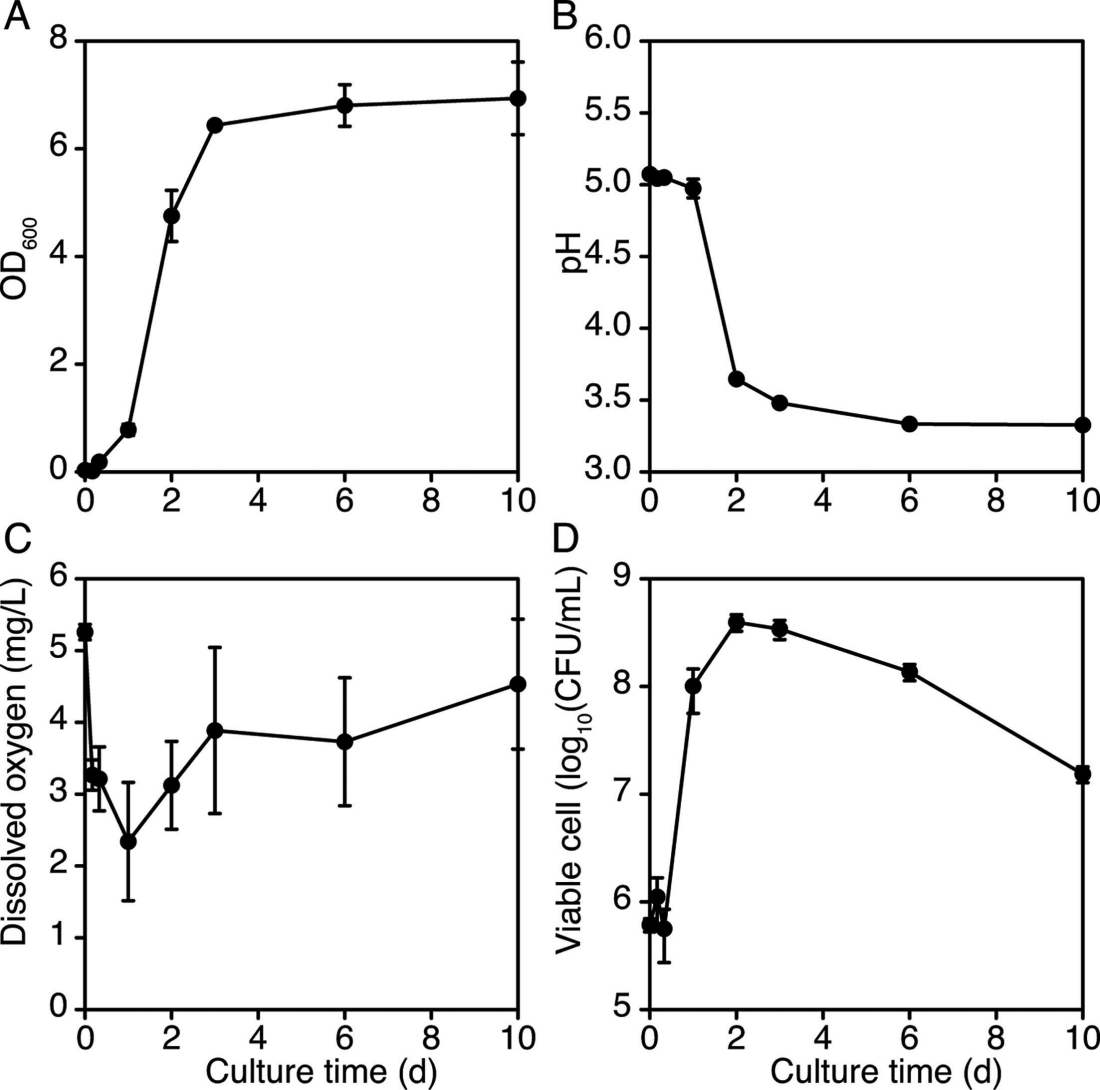
Fermentation profile using eggplant juice medium with an artificially constructed microbiota. (**A**) Growth represented by OD_600_. (**B**) pH transition. (**C**) Dissolved oxygen. (**D**) Viable cell count. Filled circle, average value of results (*n* = 3). Error bars indicate the standard deviation.

There may be various reasons for the large difference in microbial succession among the batches, as previously reported (12, 15–17). Among them, the specific effect of the initial microbiota on the dynamics was investigated by separating the initial microbiota from the raw materials, which are usually the source of the initial microbiota. The filter-sterilized EPJ was fermented with the artificially constructed microbiota to reproduce the average initial microbiota of the raw materials used in *shibazuke* production. The initial microbiota was defined based on species-level annotations derived from 16S rRNA gene V3–V4 region sequencing data (Fig. 3G through J). These results carry the risk of misannotation (23, 24), particularly within the *L. plantarum* group, which includes *L. plantarum*, *L. pentosus*, and *L. paraplantarum*, where precise identification requires *recA* gene sequences (25). Despite these limitations, this approach remains invaluable for investigating the differences in microbial succession among *shibazuke* sample sets in terms of LAB versus non-LAB, as well as acid-tolerant LAB versus acid-sensitive LAB dominance. The species satisfying the criteria (>5% relative abundance on average during the production process) for inclusion in the artificially constructed microbiota were *L. plantarum*, *Pediococcus pentosaceus*, *Vibrio palustris*, *Sphingomonas kyeonggiensis*, *Mthylobacterium phyllostachyos*, *Pyllobacterium myrsinacearum*, *Levilactobacillus brevis*, and *Marinospirillum megaterium*. However, two of these species were excluded for the following reasons. *V. palustris* required a biosafety level of 2, which could not be handled in our laboratory due to safety regulations. *M. megaterium* was detected only in the final production samples of sample sets 2 and 3 indicating this species might not be universally present in eggplant fermentation. Additionally, it was not possible to define the initial relative abundance of this species in the artificially constructed microbiota as *M. megaterium* constituted 0% of the average relative abundance of the initial microbiota. The coefficients for converting the optical density at 600 nm (OD_600_) value to the viable cell counts shown in Table 2 were used to estimate the inoculation volume of the precultured cell suspensions to construct the artificial microbiota. The LAB showed relatively similar coefficient values, while the coefficient of *P. myrsinacearum* was almost three times larger than that of the LAB, and the coefficients of *S. kyeonggiensis* and *M. phyllostachyos* were approximately 5–10 times smaller than that of the LAB. The OD_600_, pH, dissolved oxygen (DO), and viable cell count of the artificially constructed microbiota during fermentation are shown in Fig. 5. The OD_600_ and viable cell values increased exponentially from 0 h to 2 d, despite a temporary decrease in the number of viable cells at 8 h. Although the OD_600_ value remained constant from 2 to 10 d, the number of viable cells decreased by 96% during the same period. The pH value slightly decreased from 0 h to 1 d and then dropped to 3.6 at 2 d, while the DO rapidly declined from 0 h to 1 d and subsequently gradually increased. The decrease in viable cells during the later stages of fermentation is likely due to the acidic pH and nutrient depletion, while the presence of dead cells within EPJ contributed to its turbidity. The reasons for the gradual increase in DO levels remain unclear, despite the temperature being consistently controlled by the incubator and the vessels being securely fastened.

**TABLE 2:** Bacterial strains used in the artificially constructed microbiota^*a*^

Strain	Conversion coefficient (cells/OD_600_/mL)	Source
*Lactiplantibacillus plantarum* JCM1100 *Pediococcus pentosaceus* JCM5890^T^ *Levilactobacillus brevis* JCM1059^T^ *Sphingomonas kyeonggiensis* JCM18825^T^ *Phyllobacterium myrsinacearum* JCM20932^T^ *Methylobacterium phyllostachyos* NBRC105206	4.4 × 10^8^ 4.2 × 10^8^ 4.9 × 10^8^ 7.9 × 10^7^ 1.5 × 10^9^ 4.8 × 10^7^	JCM JCM JCM JCM JCM NBRC

^*a*^ JCM, Japan Collection of Microorganisms; NBRC, National Institute of Technology and Evaluation Biological Resource Center.

### Changes in the microbiota during pickle fermentation using the EPJ model

**Fig 6:**
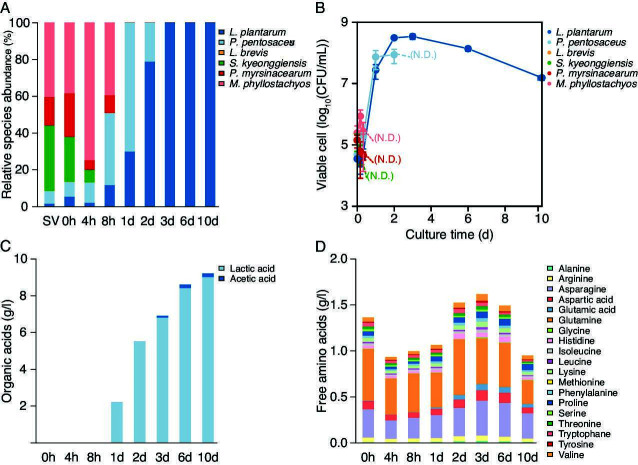
Succession of the microbiota and transition of metabolites during eggplant juice fermentation with an artificially constructed microbiota. The average values of results (*n* = 3) are shown. (**A**) Relative abundance of strains. (**B**) Viable cell count. (**C**) Average organic acid concentrations. (**D**) Average free amino acid concentrations. SV, set value based on the average initial microbiota of commercial eggplant pickles (as shown in Fig. 3B); CFU, colony-forming unit. SV, set value based on the average initial microbiota of commercial *shibazuke* pickles shown in Fig. 2F.

The succession of microbiota during EPJ fermentation using the artificially constructed microbiota is shown in Fig. 6A (average values) and Fig. S3 (for each replicate). The microbiota inoculated into the EPJ was initially dominated by non-LAB. Then, the relative abundances of *P. pentosaceus* and *M. phyllostachyos* increased, while those of other strains decreased at 4 h. The species *S. kyeonggiensis* was no longer present, and the relative abundance of *M. phyllostachyos* had decreased at 8 h. After 1 d, the microbiota consisted only of LAB. The species *P. pentosaceus* was initially dominant, comprising 70% of the microbiota after 1 d, while *L. plantarum* was dominant at 3 d. In contrast to the results from *shibazuke* pickles, no *L. brevis* was detected for up to 10 d during EPJ fermentation. Changes in the number of viable cells showed that the maximum population of *L. plantarum* was 3.9 times higher than that of *P. pentosaceus* (Fig. 5B). These microbiota dynamics were similar to the results from sample sets 3 and 4 of eggplant pickles (Fig. 3I and J). This similarity indicated that sample sets 1 and 2 (Fig. 3G and H) were subjected to accidentally inadequate conditions for pickle production.

### Correlations between viable cell counts and fermentation profiles

Correlation analysis was performed to explore the relationships between the viable population of specific species and fermentation profiles (DO and pH) to evaluate whether EPJ fermentation appropriately emulated *shibazuke* production (Table 3). The DO levels showed a positive correlation for all three aerobic species, whereas pH showed positive correlations for two aerobic bacteria, *M. phyllostachyos* and *P. myrsinacearum*. The pH was negatively correlated with two LAB, *P. pentosaceus* and *L. plantarum*. These results indicated that the EPJ fermentation was properly performed under the intended anaerobic conditions. Notably, the effect of decreasing DO on the viability of aerobic bacteria preceded the decrease in pH based on the result of DO and pH measurements in the EPJ fermentation. These results suggested that the effect of DO depletion on aerobic bacteria was primary during the initial stage of vegetable fermentation.

**TABLE 3:** Significant correlation between the species populations and the fermentation profiles from 0 h to 2 d of EPJ fermentation^*a*^

Fermentation profile	Species name	Correlation efficient
DO	*P. pentosaceus* *M. phyllostachyos* *S. kyeonggiensis* *P. myrsinacearum*	−0.61 0.53 0.67 0.60
pH	*L. plantarum* *P. pentosaceus* *M. phyllostachyos* *P. myrsinacearum*	−0.78 −0.67 0.65 0.65

^*a*^ DO, dissolved oxygen.

### Changes in the concentrations of organic acids and free amino acids during pickle fermentation using the EPJ model

The consequence of EPJ fermentation by the artificially constructed microbiota was evaluated by changes in organic acid and free amino acid concentrations. On average, the artificially constructed microbiota produced 9 g/L of lactic acid and a trace amount of acetic acid in the EPJ in 10 d (Fig. 6C average values; Fig. S4 each replicate). The increase in lactic acid corresponded with the decrease in pH. Among the amino acids measured (Fig. 6D average values; Fig. S5 each replicate), glutamic acid exhibited the highest relative increase between 0 h and 3 d, reaching more than 14 times the initial concentration. The differences observed between *shibazuke* production and EPJ fermentation, such as the detection of trace amounts of acetate and the depletion of various amino acids (arginine, histidine, and tyrosine in *shibazuke*; glycine, methionine, and tyrosine in EPJ) could be attributed to the varying dominance of LAB or to the uncertainty of species-level annotation by 16S rRNA gene V3-V4 sequencing.

### The difference in microbial succession between pickle production and EPJ fermentation

**Fig 7:**
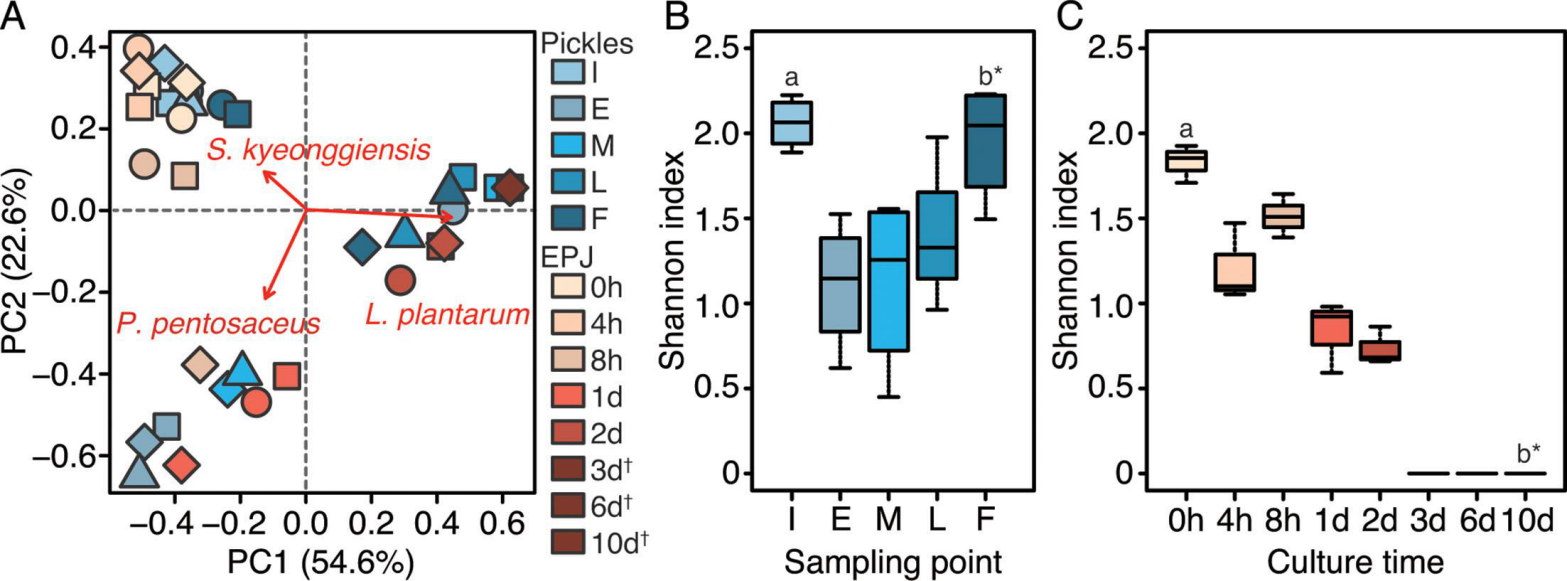
Succession of microbiota diversity during pickle and eggplant juice fermentation. (**A**) Scatter and loading plot of principal component analysis of the microbiota during pickle production and eggplant fermentation at the species level. (**B**) Shannon index during pickle production. (**C**) Shannon index during EPJ fermentation. Blue indicates pickle samples; orange indicates EPJ samples. In panel A, red arrows indicate the loading vector of the three genera that had the largest weight among the components. d, day(s); h, hour(s). I, initial production; E, early production; M, mid-production; L, late production; F, final production. †, EPJ samples from 3 to 10 d were of the same color because all plots were located in the same position. The shapes used to represent pickle samples describe the source of the samples; circle, sample set 1; square, sample set 2; diamond, sample set 3; triangle, sample set 4. The shapes used to represent EPJ samples indicate the series of experimental replicates (*n* = 3). In panels B and C, the same superscript letters indicate the pair evaluated by the Wilcoxon rank sum test. *Significant difference between samples (*P* < 0.05).

Further analysis was performed to describe the difference in microbial succession between *shibazuke* production and EPJ fermentation. The principal component analysis scatter plot provides an overview of the microbial succession (Fig. 7A). The loading plot indicated that this succession was caused by the subsequent dominance of *S. kyeonggiensis*, *P. pentosaceus*, and *L. plantarum*, although some samples from *shibazuke* production plotted a different pattern. The Shannon index (Fig. 7B and C) showed that the microbiota diversity remained relatively high during *shibazuke* production, but decreased during EPJ fermentation. The Wilcoxon rank sum test did not reveal any significant differences between initial samples from *shibazuke* production and EPJ fermentation (*P* = 0.112), but did detect differences between final samples from *shibazuke* production and EPJ fermentation (*P* = 0.044). These results suggest that it was not the initial microbiota, but rather the differing environmental conditions in *shibazuke* production and EPJ fermentation, that accounted for the variations in the final microbiota.

### Correlations between metabolite concentration and the relative abundance of microbiota

**Fig 8:**
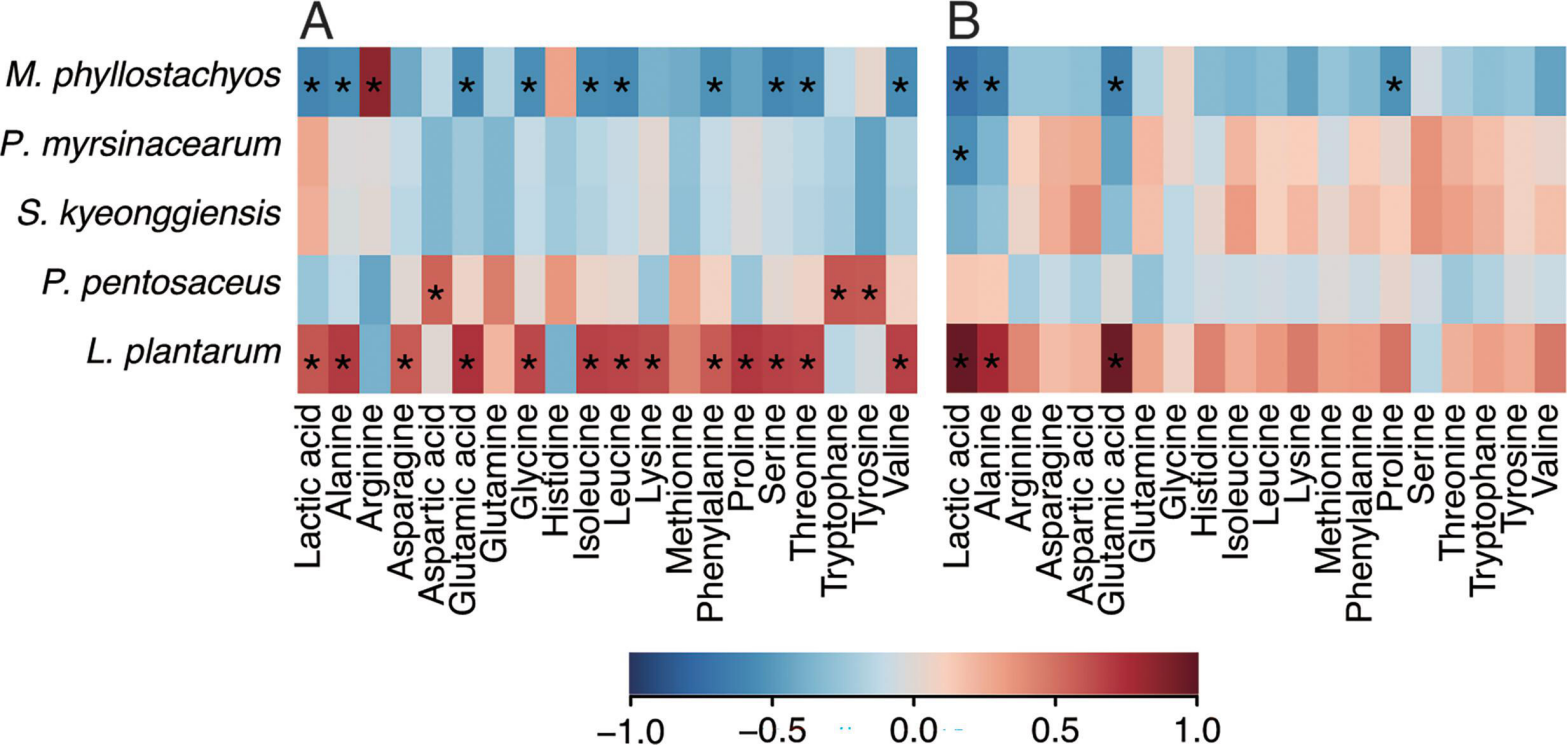
Correlation coefficient between the microbiota and metabolites. (**A**) The coefficients calculated using relative abundance in species and metabolite concentrations during *shibazuke* production. (**B**) The coefficients calculated using relative abundance in species and metabolite concentrations during EPJ fermentation. The color shows the value of the correlation coefficient. *Significant correlation (*P* < 0.05).

Considering the significant difference in alpha diversity of the final microbiota between *shibazuke* production and EPJ fermentation, the relationship between metabolites and microbes may also differ between these conditions. Therefore, correlation analysis was performed to evaluate the difference in the relationship between metabolite content and the relative abundance of microbiota at the species level (Fig. 8A, *shibazuke* production; Fig. 8B, EPJ fermentation). Despite the significant difference in alpha diversity, *L. plantarum* commonly correlated with lactic acid, alanine, and glutamic acid production during *shibazuke* production and EPJ fermentation. Although interpreting the results from *shibazuke* production at the species level requires careful consideration of the possibility of misannotation by 16S rRNA gene V3–V4 region sequences, the correlation between *L. plantarum* and several metabolites detected in EPJ fermentation suggests a potential role for *L. plantarum* in metabolite production during *shibazuke* production.

## DISCUSSION

This study aimed to investigate the effect of initial microbiota on the dynamics of the microbiota and the relationship between bacteria and metabolites produced during spontaneous eggplant fermentation. Eggplant pickles, *shibazuke*, showed two distinct patterns in microbial succession: (i) sample sets 1 and 2 (Fig. 3B, C, G, and H) showed a predominance of LAB (*P. pentosaceus* and *L. plantarum*) followed by a decrease in LAB and an increase in aerobic bacteria, and (ii) sample sets 3 and 4 (Fig. 3D, E, I, and J) showed a predominance of LAB followed by the sustained relative abundance of LAB. Observing these trends prompted further investigation into the influence of the initial microbiota on *shibazuke* production. This involved inoculating sterile EPJ with the average initial microbiota of *shibazuke* production, while controlling for temperature, salinity and anaerobicity—factors known to influence microbial succession (6, 8, 15, 16, 26). The constructed initial microbiota in the filter-sterilized EPJ showed a succession pattern similar to that of sample sets 3 and 4, with high reproducibility. The prevalence of *L. plantarum* correlated with the production of lactic acid, alanine, and glutamic acid during *shibazuke* production and EPJ fermentation.

The microbial succession in *shibazuke* sample sets 3, 4 and fermented EPJ suggested that the same initial microbiota underwent the same interactions, resulting in the same microbial succession under *shibazuke* production conditions. Furthermore, these successions were essentially equivalent to those in spontaneously fermented cereal beverage (27), sauerkraut (8), cucumber (28), and kimchi (6), characterized by a dominance of nomadic LAB (i.e., *L. plantarum*) (13), which can thrive in various environments with resilience to high acidity and salinity-induced stress. On the contrary, *shibazuke* sample sets 1 and 2 having equivalent initial microbiota to EPJ underwent the disrupted dominance of LAB, similar to previous studies (9–12). The disrupted microbial succession appeared to have been induced by relatively aerobic conditions, as evidenced by the substantial abundance of aerobic bacteria. Sampling can be one of the reasons that pickles were accidentally exposed to oxygen from open air atmosphere, resulting in the growth of aerobic bacteria that deviated the microbiota from the standard succession. Therefore, the impact of initial microbiota on microbial succession should be considered less significant than anaerobicity.

The results from EPJ fermentation facilitate the understanding of the mechanisms behind the standard microbial succession during *shibazuke* production. The initial decrease in aerobic species, such as *S. kyeonggiensis and P. myrsinacearum*, was triggered by the reduction in DO, observed before the decline in pH. This finding highlights the importance of creating anaerobic conditions to swiftly eliminate unwanted aerobic bacteria and promote lactic acid fermentation. Subsequently, a slight decrease in pH was proven to be sufficiently detrimental to *P. myrsinacearum* and *M. phyllostachyos.* Following this, the population of *P. pentosaceus* increased, further lowering the pH through lactic acid production. Eventually, *P. pentosaceus* and *L. plantarum* survived, depending on their acid-tolerance abilities. The final decrease in *L. plantarum* is presumed to be due to excessive acidity, as reported previously (27). The correlation and alpha-diversity analysis indicated that *L. plantarum* plays an important role in the production of lactic acid, alanine, and glutamic acid, regardless of the dynamics of the microbiota (Fig. 6 and 7). While *L. plantarum* is known as an alanine autotroph (29), genes related to alanine biosynthesis from precursors, such as glutamine, asparagine, or pyruvate, remain unidentified according to the KEGG database (accessed Nov. 9, 2023) (30). It remains unclear whether *L. plantarum* produced alanine through biosynthesis or other reactions, e.g., protein digestion, under *shibazuke* production conditions. Glutamic acid, contributing to umami taste, can be produced by glutaminase, glutamine synthase from glutamine (31), or by glutamate dehydrogenase from 2-ketoglutarate (32, 33). However, genes of glutaminase and glutamine synthase remain unidentified in most strains of *L. plantarum*, and 2-ketoglutarate was not detected from the tested samples. The increase in glutamic acid was likely obtained by protein digestion by extracellular proteases (34) or by spontaneous deamidation of glutamine under acidic conditions (35). The reduction in total amino acid could indicate the deactivation of excreted proteases from *L. plantarum* due to excessive acidity, while the consumption of amino acids by *L. plantarum* remained active to the final timepoint. It should be noted that the roles attributed to *L. plantarum* might also apply to other species of the *L. plantarum* group, such as *L. pentosus* and *L. paraplantarum*, in *shibazuke* production, as the 16S rRNA gene V3–V4 sequencing cannot distinguish these species (25).

Although the general trends of microbial succession in *shibazuke* sample sets 3 and 4 were equivalent to EPJ fermentation, some discrepancies were observed concerning *L. plantarum* prevalence, *L. brevis* detection, total organic and free amino acids levels, and their composition. These differences can be attributed to factors other than the initial microbiota, such as the survival of stress-sensitive species and the disparity in carbon and nitrogen sources from the raw material. First, 16S rRNA gene analysis detected minor species alongside *L. plantarum* from the final microbiota in *shibazuke*, whereas viable cell counts only detected *L. plantarum* at the final EPJ fermentation. As previously suggested, 16S rRNA gene sequence might detect unexpectedly stable DNA from dead cell (14). However, stress-sensitive species, including minor species, likely survived resulting in a difference in *shibazuke* microbiota, and subsequently, it altered the organic and amino acid compositions. The decrease in lactic acid in sample sets 1 and 2 of *shibazuke* also can be due to the survived species utilizing lactic acid as previously reported in cucumber pickles (36). The survival of stress-sensitive species, also observed in sample sets 1 and 2, could be attributed to the small ditches on the surface of sliced eggplant functioning as shelters, similar to pores and cracks on the surface of wooden barrels used for Lambic fermentation that allow microorganisms to form protective biofilms against environmental stresses (37). However, this sheltering possibility raises concerns about the survival of undesirable microorganisms, posing a risk to food safety. In the pickles tested, the potential pathogenic bacteria, *V. palustris* and Enterobacteriaceae bacteria, were found. Previously, appropriate kimchi production conditions have been shown to reduce the risk of food poisoning from the pathogenic *Echerichia coli* and *Salmonella* (38). The standard *shibazuke* production conditions, where pH was lower than 4.0, should also impair the growth of *V. palustris* and Enterobacteriaceae bacteria, owing to their sensitivity to low pH ensuring food safety (27, 39). The quantity of carbon and nitrogen sources from raw material influenced the difference in the total content of organic and amino acids. The use of a whole eggplant in *shibazuke* production provides insoluble content associated with carbon and nitrogen sources, but it was removed from EPJ by filter-sterilization. The total amount of organic and free amino acids was reduced in EPJ fermentation due to substrate limitation, and consequently, it can curtail the growth of *L. brevis*, which requires most of the amino acids for growth (40), due to the lack of the necessary amino acids. This suggestion is further supported by the result of acetic acid detection from EPJ fermentation because *L. plantarum* is known to produce acetate from lactate under nutrient-depleted conditions (41).

In the present study, the dynamics of microbiota and metabolite contents were investigated using *shibazuke* pickles and EPJ. Modeling the microbial succession with an artificially constructed microbiota in sterile EPJ revealed the high reproducibility of microbial succession and interaction originating from the initial microbiota during *shibazuke* production. This study also established the robust correlation with *L. plantarum* and the production of lactic acid, alanine, and glutamic acid. Modeling the microbial ecosystem offers a promising approach to understanding the complex interactions and functional roles of microbes, thereby contributing to the development of safe and delicious fermented foods (42). The methodologies employed in this study constructed a microbial ecosystem that included undesired microbes, demystifying microbial succession through controlled experiments. This approach can be extended to other raw materials, enhancing our understanding of microbial communities in fermented vegetables and potentially improving pickle production in the future.

## MATERIALS AND METHODS

### Sample collection and processing

Samples of the commercial eggplant pickle, *shibazuke*, produced via spontaneous fermentation, were provided by three manufacturers (A, B, and C) from Ohara district in Kyoto, Japan, in August and September 2017. Each manufacturer produced *shibazuke* pickles using their own procedures, but general production procedures are as follows. The pickles were prepared from sliced eggplant (*Solanum melongena*), flavored and colored with perilla herbs (*Perilla frutescens* var. *crispa*). The eggplant slices and perilla herbs underwent brief pre-dehydration with salt. Subsequently, the pre-dehydrated vegetables were fully packed with salt yielding initial pickling brine in the fermentation containers (75 L) and hermetically sealed and pressed by a weight stone (80 to 100 kg), eliminating any residual air to facilitate anaerobic fermentation. The pickles were fermented without temperature control. Approximately 50 g of pickles was sampled by the manufacturers under open air conditions at various time points: d 0 (initial production), 3 or 5 d (early production), 7 d (mid-production), 14 d (late production), and 30 d (final production) (Table 1). The samples from early, mid-, and late production stages were taken from the pickles under the brine near the surface to avoid disturbing the commercial pickle production process. Samples from the final production were randomly taken from the container. Additionally, 50 mL of pickling brine from the initial production was obtained directly from the fermentation container. Except for the initial production, brine samples were obtained by squeezing the brine from the corresponding pickle samples. Pickle and brine samples were stored in a food-grade plastic bag and in a 50-mL sterilized tube, respectively. All samples were promptly stored at 4°C before being sent to our laboratory. The samples were transported within 2 d, maintaining a temperature range between 0 and 12°C. Manufacturer A provided two batches of samples because the pickles were produced twice (August and September) in 2017. Final production by Manufacturer C was completed in 14 d; therefore, the 14-d sample from this manufacturer was processed as the final production sample in the study. The results from multiple samples taken at the same time point from the same batch were used to calculate averages in the following analyses (Table 1). Bacterial cells for DNA extraction were collected from all samples using the following procedure. First, pickles were aseptically cut to approximately 140 cm^2^ and washed by shaking vigorously in sterile 0.85% NaCl solution (10 mL) for 15 s. Debris, such as broken eggplant and herb leaves, in the washing solution was removed using a filter (pore size, 40 µm). The filtered solution was then centrifuged at 8,000 × *g* for 10 min at 4°C, after which the supernatant was discarded, leaving the bacterial cell samples. Cells were collected from brine samples using the same method. All cell samples were stored at −20°C until the DNA was extracted. Following cell collection, the remaining pickle and brine samples were stored at −20°C until further analysis.

### DNA extraction and 16S rRNA gene sequencing

The collected cells were resuspended in a Tris–EDTA buffer containing lysozyme (1 g/L) and incubated at 37°C for 5 min. DNA was then extracted from the processed cells by bead-beating using a Quick-DNA Fungal/Bacterial Kit Miniprep (Zymo Research, Irvine, CA, USA). The V3–V4 region in the 16S rRNA gene of the extracted DNA was amplified by PCR using universal primers (forward, 5′-CTA CGG GGG GCA GCA G-3′; reverse, 5′-GGA CTA CCG GGG TAT CT-3′; expected amplified region, 465 base pairs) following barcode and adaptor sequences (forward, 5′-AAT GAT ACG GCG ACC ACC GAG ATC TAC ACT CTT TCC CTA CAC GAC GCT CTT CCG ATC T-3′; reverse, 5′-CAA GCA GAA GAC GGC ATA CGA GAT [index sequences (six bases)] GTG ACT GGA GTT CAG ACG TGT GCT CTT CCG ATC T-3′) (43) of Illumina MiSeq (Illumina, San Diego, CA, USA) as previously described (44). The size and concentration of the amplified DNA were determined using an Agilent DNA 1000 Kit and an Agilent 2100 Bioanalyzer (Agilent Technologies, Santa Clara, CA, USA), after which, equimolar amounts of DNA from each sample were loaded into a MiSeq sequencer.

### Processing 16S rRNA gene sequence data for microbiota analysis

The reads from Illumina MiSeq analysis were processed as previously described (44). Briefly, reads containing undetermined base(s), i.e., N(s), and *phiX* sequences were removed by bowtie2 (version 2.1.0) (45). Unpaired reads were deleted using USEARCH (version 7) (46). Processed reads were filtered, denoised, and then merged with dada2 (47) of the R program (version 1.8). Subsequently, an ASV table was constructed from the merged reads. The chimeras were removed, after which, the ASV table and reads were imported into Qiime2 (48) (version 2018.11) for further analysis. Taxonomy at the genus level was assigned to the ASVs using the Greengenes (version 13.8) database (49). ASVs annotated to the mitochondria and chloroplasts were removed, after which, all reads from each sample were used for further analysis. The genera and species for which the average relative abundance in the whole process was less than 1% were consolidated to “Others.” Additional Basic Local Alignment Search Tool searches with the National Center for Biotechnology Information database (accessed Sept. 17, 2020) were used to identify top-hit species assigned to the ASV that the Greengenes database could not classify at the species level.

### Analytical methods for salinity, pH and metabolites

Brine samples from the initial product, as well as squeezed juice from the early to the final production phase, were used to measure salinity and pH. Salinity was measured using a LAQUAtwin-Salt-22 meter (Horiba, Kyoto, Japan). Samples were prepared for the measurement of organic acids and free amino acids using the following procedures. *Shibazuke* pickles (approximately 10 g) were chopped roughly, after which, 1 g of the chopped sample was homogenized with the same weight of ultrapure water using a high-power homogenizer (ASONE, Tokyo, Japan) at 3,000 rpm for 5 min on ice. The homogenized juice and pickling brine were subsequently centrifuged at 20,400 × *g* and 4°C for 5 min to obtain the supernatant. Organic acids in the supernatants were analyzed using high-performance liquid chromatography (HPLC) using a Chromaster system (Hitachi, Tokyo, Japan) equipped with an Aminex-HPX87H column (BioRad Laboratories, Hercules, CA, USA). The conditions for HPLC were based on the instruction of column manufacture as follows: mobile phase, 4 mM sulfuric acid; flow rate, 0.5 mL min^−1^; column over temperature, 40°C; running time, 30 min; detection, UV 210 nm. The measured compounds in the sample were quantified using external standard solution consisting of 0.5 g/L of 2-ketoglutarate, 0.5 g/L of pyruvate, 10 g/L of succinate, 10 g/L of acetate, 10 g/L of propionate, 10 g/L of butylate, 5 g/L citrate and 5 g/L malate. The samples were diluted to make the signals lower than the external standards. Amino acids in the supernatants were analyzed by UPLC using a NexeraX2 system (Shimadzu, Kyoto, Japan) equipped with a Shim-pack Velox C18 column (Shimadzu), following the manufacturer’s specified conditions. Prior to analysis, the amino acids in the samples were derivatized using o-phthalaldehyde, 3-mercaptopropionic acid, and 9-fluorenylmethyl chloroformate as described previously (50). The conditions for UPLC were as follows: the mobile phase consisted of solution A (20 mM potassium phosphate) and solution B (45% acetonitrile and 40% methanol in MilliQ water); flow rate was set at 0.65 mL min^−1^ in gradient mode. The gradient program was 89.5% solution A from 0 to 1.5 min, 89% solution A from 1.5 to 6 min, 78% solution A from 6 to 8 min, 70% solution A from 8 to 10.5 min, 47% solution A from 10.5 to 12.5 min, 100% solution B from 12.5 to 17 min. The column oven temperature was maintained at 35°C. The detection was performed using a fluorescence detector with excitation at 350 nm and emission at 450 nm for primary amino acids (0–10.8 min), and excitation at 266 nm and emission at 305 nm for secondary amino acids (10.8–17 min). The measured compounds in the sample were quantified using external standards using Amino Acids Mixture Standard Solution Type H (FUJIFILM Wako Pure Chemical Corporation, Osaka, Japan) diluted to the upper limit of detection (125 µM) in 0.01 M hydrochloric acid as well as separately prepared 125 µM solution of asparagine, glutamine and tryptophan in MilliQ water.

### EPJ model of *shibazuke* fermentation using an artificially constructed microbiota

#### EPJ preparation

EPJ was prepared as shown in Fig. 1, a modified procedures previously reported using cucumbers (51), and was then used as the raw material for emulated *shibazuke* production using the artificially constructed microbiota. Eggplants obtained from a local market were finely chopped and wrapped in a straining cloth before squeezing. The squeezed juice was then centrifuged at 8,000 × g for 20 min at 20°C. Next, the supernatant from the juice was prefiltered using glass-fiber filter paper (pore size, 0.4 µm). NaCl was then added to the filtered juice to give a salinity of 3.6%, which was the average final salinity of the commercial *shibazuke* pickles measured in this study. Finally, the prepared juice was sterilized by filtration using a membrane filter (pore size, 0.4 µm). The sterile EPJ was then stored at ˗20°C until use.

### Cell preparation and coefficient determination for converting OD to viable cell count in artificial microbiota construction

The six bacterial strains (Table 2) are used as the artificially constructed microbiota to ferment EPJ. Species with an average relative abundance of more than 5% during commercial production for at least one sampling point were selected for the artificially constructed microbiota based on the assumption that major bacteria had a larger influence on pickle fermentation. The selected six species, *Lactiplantibacillus plantarum* JCM1100, *Pediococcus pentosaceus* JCM5890^T^, *Levilactobacillus brevis* JCM1059^T^, *Sphingomonas kyeonggiensis* JCM18825^T^, *Phyllobacterium myrsinacearum* JCM20932^T^, and *Methylobacterium phyllostachyos* NBRC105206, were cultivated from a frozen stock on an agar plate of the following preculture media for 3 d: Man-Rogosa and Sharpe (Difco Lactobacilli MRS Broth, Becton and Dickinson, Franklin Lakes, NJ, USA) for *L. plantarum*, *P. pentosaceus*, and *L. brevis*; modified Tryptic Soy Broth (TSB) [peptone 15 g, Bacto Soytone 5 g (Becton and Dickinson), and sodium chloride 5 g in 1 L of water, adjusted to pH 7.0 by NaOH] for *S. kyeonggiensis* and *P. myrsinacearum*; and Methylobacterium Medium [peptone 10 g, yeast extract 2 g, MgSO_4_•7H_2_O 1 g, and methanol 5 mL in 1 L of water, adjusted to pH 7.0 by NaOH] for *M. phyllostachyos*. Agar (15 g/L) was added when necessary. The cells grown on agar plates were subsequently cultured with the same preculture medium described above for 18 h at 30°C. The initial OD_600_ was 0.01 for *L. plantarum*, *L. brevis*, *P. pentosaceus*, *S. kyeonggiensis*, and *P. myrsinacearum*, while it was 0.1 for *M. phyllostachyos*. The coefficient for converting OD_600_ to a viable cell count was determined using the OD_600_ and CFUs of the precultured broth. The CFUs were measured by plating the preculture broth at an appropriate dilution onto modified TSB agar plates in duplicate. The preculture broth of each strain for CFU counting was prepared independently in at least triplicate. The OD_600_ measurement was conducted using a spectrophotometer on samples that had been appropriately diluted to achieve values less than 0.4. The cells for EPJ fermentation were collected by centrifuging the preculture broth of each strain at 8,000 × g for 10 min at 20°C. The cells were then washed with sterile 0.85% NaCl solution twice, after which they were resuspended in sterile 0.85% NaCl solution and used for EPJ fermentation.

### EPJ fermentation with the artificially constructed microbiota

**Fig 9:**
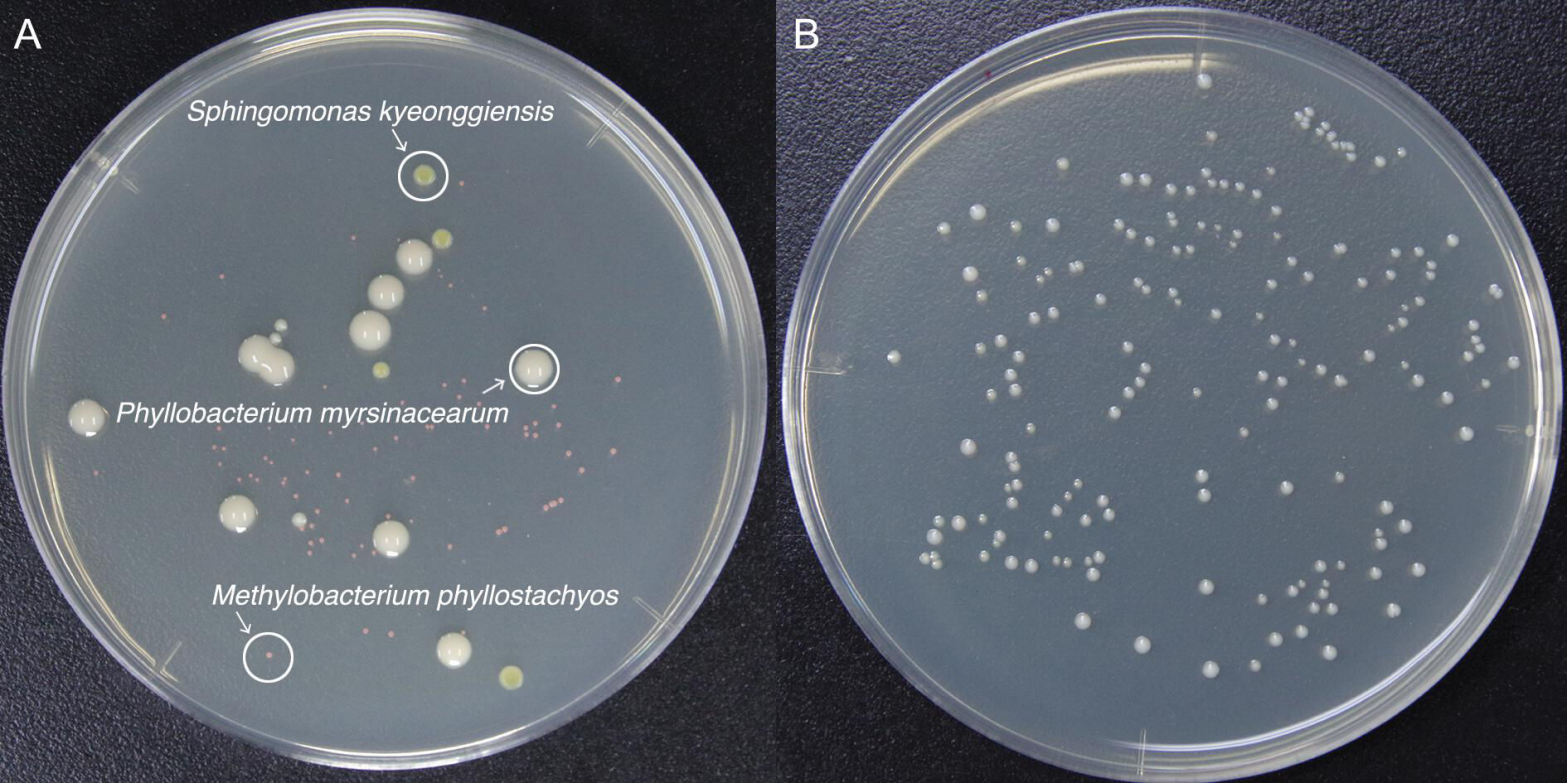
Colonies of the strains used in EPJ fermentation. Tryptic soy broth plates incubated for 3 days. The viable cell count of *Sphingomonas kyeonggiensis*, *Phyllobacterium myrsinacearum* and *Methylobacterium phyllostachyos* was determined on the basis of colony morphology. (**A**) inoculation of culture broth from 4 hour containing *S. kyeonggiensis*, *P. myrsinacearum* and *M. phyllostachyos* and some lactic acid bacteria; (**B**) inoculation of culture broth from 1 day containing *Lactiplantibacillus plantarum* or *Pediococcus pentosaceus*.

The prepared cells were inoculated into the EPJ to construct the artificial microbiota based on the average composition of selected species at the initial point of production in the commercial *shibazuke* pickles. Inoculation volume was determined as follows: the OD_600_ of a diluted cell suspension of each species was measured to estimate viable cell content using the conversion coefficient described in the previous section. The required volume of the cell suspension for each species was then calculated based on their respective viable cell content to achieve the targeted relative abundances, which were as follows: *L. plantarum*, 1.6%; *P. pentosaceus*, 6.8%; *L. brevis*, 0.1%; *S. kyeonggiensis*, 35.6%, *P. myrsinacearum*, 15,4%; *M. phyllostachyos*, 40.5% [as shown in Fig. 6A as a set value (SV)]. Subsequently, the cell suspensions of each species were mixed according to the calculated volume ratio. Finally, the mixed suspension was inoculated into EPJ to achieve a total OD_600_ of 0.01. A sterile 250 mL glass bottle was employed as a closed fermentation vessel, featuring an inlet connected to a gas bag filled with nitrogen gas, a sampling port, and an exhaust port with a non-return valve. The 250 mL of sterile EPJ were poured into the fermentation vessel with a stirrer bar. After inoculation, nitrogen gas was injected to purge air from the bottle headspace, providing an anaerobic atmosphere. Fermentation of the EPJ was performed statically at 28°C, maintained by an incubator. The EPJ was only stirred moderately before sampling. Culture broth samples were taken at 0, 4, and 8 hours (h) and at 1, 2, 3, 6, and 10 d after inoculation to use for OD_600_, pH and following analysis. The samples of 5 mL were drawn using a syringe via the sampling port, with nitrogen gas concurrently introduced into the vessel from the gas bag to maintain an anaerobic atmosphere. The sampling time was determined based on the preliminary experiment to detect changes in the microbiota and viable cell count. The DO was measured using a DO-1000PE sensor (CUSTOM Corporation, Tokyo, Japan). The 100 µL of sampled culture broth appropriately diluted by sterile 0.85% NaCl solution was also inoculated onto a modified TSB agar plate to determine the number of CFUs on plates with at least 100 colonies for samples taken between 0 and 8 h and at least 50 colonies for samples taken between 1 and 10 d. *S. kyeonggiensis*, *P. myrsinacearum*, and *M. phyllostachyos* on the CFU count plates were identified by colony appearance owing to their clearly distinguishable features, which are as follows: *S. kyeonggiensis* colonies are bright yellow; *P. myrsinacearum* colonies are white, shiny, and slimy in appearance; and *M. phyllostachyos* colonies are pink (Fig. 9A). The other species (*L. plantarum*, *P. pentosaceus*, and *L. brevis*) were identified by random amplified polymorphic DNA (RAPD) PCR using RAPD analysis primer 3 (5′- GTA GAC CCG T −3′) from the Ready-To-Go RAPD PCR kit (GE Healthcare, Chicago, IL, USA) according to the manufacturer’s instructions because they were indistinguishable (Fig. 9B). The CFU count was used to calculate the viable cell count in 1 mL of culture broth and to estimate the relative abundance of each strain. To determine the metabolite concentrations, culture broth was centrifuged at 20,400 × g and 4°C for 5 min, after which the supernatant was analyzed by HPLC and UPLC as described above. The EPJ was fermented by the artificially constructed microbiota independently prepared in triplicate.

### Statistical analysis

Correlation coefficients between the fermentation profile (pH and DO) and estimated viable cell count of each species during EPJ fermentation were calculated using Pearson’s method. This calculation of the correlation coefficients used the data obtained from 0 h to 2 d when the total viable cell counts were increasing. The correlations between relative abundance and metabolite production during *shibazuke* production and EPJ fermentation were calculated using the same method. Alpha diversity was evaluated by calculating the Shannon index (52), after which significant differences in alpha diversity between pickle and EPJ samples were identified using the Wilcoxon rank sum test with the pair of initial samples (pickles, initial production samples; EPJ, 0 h samples) or the pair of final samples (pickles, final production samples; EPJ, 10 d samples). Principal component analysis was performed based on the relative abundance of species during pickle production and EPJ fermentation. All statistical analyses were conducted using R (version 3.5.1). A *P* value < 0.05 was considered to indicate significance for all statistical analyses.

## Data Availability

The datasets generated during sequencing by Illumina Miseq are available in the DNA Data Bank of Japan (DDBJ) repository under accession number DRA013234. The other data generated or analyzed during this study are included in this published article and its supplemental materials.

## SUPPLEMENTAL MATERIAL

Supplemental figures spectrum.00464-24-s0001.docx Fig. S1-S5.

ASM does not own the copyrights to Supplemental Material that may be linked to, or accessed through, an article. The authors have granted ASM a non-exclusive, world-wide license to publish the Supplemental Material files. Please contact the corresponding author directly for reuse.
