# Supplementary material for: Influence of the initial microbiota on eggplant shibazuke pickle and eggplant juice fermentation
Source: Microbiol Spectr. 2024 Jul 17;12(8):e00464-24. doi: 10.1128/spectrum.00464-24 (PMC11302249; doi:10.1128/spectrum.00464-24)
Supplement: Supplemental figures — Fig. S1-S5. [file spectrum.00464-24-s0001.docx]

**Supplemental materials**

Supplemental Figures

**Fig. S1 Change in lactic acid content in each commercial *shibazuke* pickle sample**

(A) sample set 1; (B) sample set 2; (C) sample set 3; (D) sample set 4. I, initial production (0 day [d]); E, early production (3 d or 5 d); M, mid-production (7 d); L, late production (14 d); F, final production (30 d). The late production sample of sample set 3 was used as the final production sample as described in the manuscript.

**Fig. S2 Change in free amino acid concentrations in each commercial *shibazuke* pickle sample**

(A) sample set 1; (B) sample set 2; (C) sample set 3; (D) sample set 4. I, initial production (0 day [d]); E, early production (3 d or 5 d); M, mid-production (7 d); L, late production (14 d); F, final production (30 d). The late production sample of sample set 3 was used as the final production sample as described in the manuscript.

**Fig. S3 Dynamics of relative abundance of species in each culture of eggplant juice medium fermentation**

Panels (A) to (C) indicate the results from independent culture. h, hour(s); d, day(s).

**Fig. S4 Changes in organic acid contents in each culture of eggplant juice medium fermentation**

Panels (A) to (C) indicate the results from independent culture. h, hour(s); d, day(s).

**Fig. S5 Changes in free amino acid contents in each culture of eggplant juice medium fermentation**

Panels (A) to (C) indicate the results from independent culture. h, hour(s); d, day(s).
